# “*Mens Sana in Corpore Sano*”: The Emerging Link of Motor Reserve with Motor and Cognitive Abilities and Compensatory Brain Networks in SCA2 Patients

**DOI:** 10.3390/biomedicines10092166

**Published:** 2022-09-02

**Authors:** Libera Siciliano, Giusy Olivito, Nicole Urbini, Maria Caterina Silveri, Maria Leggio

**Affiliations:** 1 Department of Psychology, Sapienza University of Rome, 00185 Rome, Italy; 2 Ataxia Laboratory, Fondazione Santa Lucia IRCCS, 00179 Rome, Italy; 3 Department of Psychology, Catholic University of the Sacred Heart, 20123 Milan, Italy

**Keywords:** motor symptoms, cerebellum, compensation mechanisms, spinocerebellar ataxia type 2, NBS, functional connectivity

## Abstract

The ability to resiliently cope with neuropathological lesions is a key scientific concern. Accordingly, this study aims to investigate whether motor reserve (MR), likely to be boosted by exercise engagement in a lifetime, affects motor symptom severity, cognitive functioning, and functional brain networks in spinocerebellar ataxia type 2 (SCA2)—a cerebellar neurodegenerative disease. The MR of 12 SCA2 patients was assessed using the Motor Reserve Index Questionnaire (MRIq), developed ad hoc for estimating lifespan MR. The International Cooperative Ataxia Rating Scale was used to assess clinical motor features, and neuropsychological tests were used to evaluate cognitive functioning. Patients underwent an MRI examination, and network-based statistics (NBS) analysis was carried out to detect patterns of functional connectivity (FC). Significant correlations were found between MRIq measures and the severity of motor symptoms, educational and intellectual levels, executive function, and processing speed. NBS analysis revealed a higher FC within subnetworks consisting of specific cerebellar and cerebral areas. FC patterns were positively correlated with MRIq measures, likely indicating the identification of an MR network. The identified network might reflect a biomarker likely to underlie MR, influenced by education and cognitive functioning, and impacting the severity of motor symptoms.

## 1. Introduction

The concept of reserve, first introduced as the possibility of the brain being enriched and made resilient to neuronal damage by an individual’s engagement in experiences throughout their lifespan, is increasingly gaining attention from the scientific community and given different connotations [1,2]. Brain reserve is considered to be morphological and quantitative since it is attributed to the remaining intact neurons/dendrites/synapses after damage [3]. Cognitive reserve refers to the notion that efficient pre-existing cognitive functioning allows one to better handle deterioration later in life or due to brain damage [1]. As an epitome of the two previous concepts, the neural reserve refers to brain network functioning, i.e., to the efficiency of varying neural circuits to engage in proper information processing and functioning despite brain deterioration [1]. Such mechanisms are supposed to result in an individual’s ability to cope with neuropathological lesions in a resilient way [3]. In the field of neurodegenerative diseases, increasing attention is being paid to compensatory mechanisms with regard to brain structure and networks, along with pre-existing and enhanced cognitive abilities. Being first accounted for in Alzheimer’s disease, cognitive reserve is now widely accepted to influence both the onset and severity of symptoms [1]. Along with the interest in cognitive reserve, importance is lately being attributed to motor reserve (MR), although the literature on this topic is very scarce [4,5]. However, premorbid exercise engagement and cognitive factors expressed in terms of years of education are significant predictors for motor performance in Parkinson’s disease (PD), indicating a role for both educational attainment and physical exercise in enhancing motor reserve and enabling resilient coping with the pathology [4,6]. A recent study using network-based statistics (NBS) analysis identified an MR network in PD involving the basal ganglia, inferior frontal cortex, insula, and cerebellar vermis [7]. An enhanced degree of functional connectivity (FC) within nodes of the MR network was indicative of greater MR, since it correlated well with educational attainment, and was associated with a slower rate of longitudinal increase in dopaminergic medication doses during a 2-year follow-up period. Likewise, increased connectivity in the default-mode and cortical–cerebellar networks has been proposed to play a role in postponing symptom expression and compensating for the effects of relentless neurodegeneration in amyotrophic lateral sclerosis [8,9]. In this framework, a necessary remark needs to be made with regard to the cerebellum. The notion of cerebellar reserve has been recently and elegantly examined by Mitoma et al. [3], highlighting the sophisticated capacity of the cerebellum to compensate for and restore function in response to both cerebellar focal and degenerative insults. The hypothesis of brain network rearrangement driven by cerebellar activity exerted on cortical and subcortical brain regions is supported by the nature of cerebellar structure and functions—it contains most of the neurons in the brain; it has a uniform cytoarchitecture throughout the cerebellar cortex; its topographical arrangement in functional microzones enables reciprocal anatomical and functional connections with many extracerebellar regions; and through its functional units it enables the acquisition and detection of internal models linked to information learning and prediction in diverse functions (i.e., perceptive, motor, cognitive), in turn guaranteeing the optimisation and updating of those internal models even when cerebellar damage occurs, and in concert with cerebellar synaptic plasticity [3]. In support of the notion of neural reserve defined in terms of cerebral and cognitive/motor compensatory mechanisms, a recent study conducted on patients with spinocerebellar ataxia type 2 (SCA2) revealed that patterns of changes in hyperconnectivity between specific cortical areas were associated with better performance in tasks evaluating motor, learning, and attentional functions [10]. SCA2 is a rare autosomal dominant inherited cerebellar neurodegenerative disease caused by the presence of expanded CAG trinucleotide repeats (≥32 CAG) in the gene encoding ataxin-2 [11]. CAG expansion size influences the onset of the disease and the rate of progression in SCA2 [12]. Hence, the larger the expansion, the earlier the onset and the worse the clinical picture [12]. In general, the primary clinical signs consist of cerebellar manifestations such as ataxia of gait/posture/limbs, dysmetria, dysarthria, hypotonia, action tremor, and adiadochokinesia [12]. Although the first occurring symptoms are related to motor control and coordination, recent literature has provided thorough evidence that cognitive and social–emotional deficits develop along with the progression of the disease, impacting the whole quality of life of patients and caregivers [13,14,15,16]. Consistent with functional deterioration, the earliest sign of the degenerative progression in SCA2 is characterised by deterioration in the olivopontocerebellar system [17,18], along with damage to the cerebellar white (WM) and grey matter (GM), affecting the cerebellar hemispheres and vermis, as well as the thalamus, pons, and mesencephalon [12], followed by extensive GM reduction in several supratentorial areas [19,20]. Consensus has also been reached about the presence of decreased FC within cerebellar–cortical circuits, likely caused by the primary neurodegeneration of specific cerebellar units [15,16]. Such a prominent action of the cerebellum on distal cerebral regions is known to impact a wide range of functional domains in SCA2, preventing the fluid and optimised cerebellar control crucial for the proper implementation of motor, attentional, executive, emotional, and social behaviours [10,16,20]. At the same time, as mentioned above, patterns of changes in hyperconnectivity between cortical areas of the default-mode and frontoparietal networks have also been described in SCA2 patients and attributed to compensatory mechanisms [10]. Nevertheless, to date, no study has been conducted with the aim of deepening the neural and motor reserves in SCA2, or in cerebellar neurodegenerative diseases in general. Therefore, in the present study, we aimed to investigate MR in SCA2 patients and its association with both motor and cognitive performance, and with neural reserve, in terms of compensatory mechanisms driven by functional hyperconnectivity. In particular, cerebellar and cerebral internodal hyperconnectivity were investigated by applying a whole-brain analysis driven by graph theory—a mathematical approach that describes complex systems as networks [21,22]. Specifically, increased FC patterns were investigated by means of NBS—a method that allows the identification of connections and subnetworks representing the brain by a graph constituted by a number of regions named nodes, which are functionally coupled to one another by edges.

## 2. Materials and Methods

### 2.1. Participants

Twelve patients with a genetic diagnosis of SCA2 (female/male: 7/5; mean age/SD at the time of the clinical and magnetic resonance imaging (MRI) assessment: 48.3/8.3 (years); mean educational level/SD: 14.4/3.7 (years) were recruited from the Ataxia Laboratory of the IRCCS Santa Lucia Foundation. At the time of evaluation, the disease duration from the date of the formal diagnosis was longer than 6 months for all patients. The neurological examination carried out by an expert neurologist showed that all patients presented with pure cerebellar motor syndrome. No other neurological signs were detected except for CB4, who showed a Babinski sign. Apart from CB11, who presented with a coronary stent incompatible with MRI scanning, all other patients underwent an MRI examination, and had no macroscopic extracerebellar brain abnormalities, as assessed by an expert neuroradiologist by visual inspection of the T2-weighted MRI scans. Some of these patients had participated in previous studies [15,16,23]. The demographic and clinical characteristics of the patients are reported in Table 1. A control group was used for the MRI analysis, and was based on retrospective MRI data of healthy participants collected from 2014 to 2019 at the Neuroimaging Laboratory of the Santa Lucia Foundation. This group was composed of 24 healthy subjects (HS) (mean age/SD: 41.5/13.6; mean education level/SD: 15.5/2.9) with no history of neurological or psychiatric illness. Statistical analysis revealed no significant differences between SCA2 patients and healthy subjects in terms of mean age (*t* test: *t* = −1,47; *p* = 0.151) or mean education level (*t* test: *t* = 0.45, *p* = 0.66). The sample size of HS was based on a previous study in which MRI data were analysed and compared between SCA2 patients and healthy controls by means of NBS analysis [15]. This study was approved by the Ethics Committee of the Santa Lucia Foundation, in accordance with the principles expressed in the Declaration of Helsinki. Written informed consent was obtained from each subject.

### 2.2. Motor Reserve

MR was assessed by means of the Motor Reserve Index Questionnaire (MRIq) [24,25]—a questionnaire developed ad hoc for estimating indices of acquired motor reserve during an individual’s lifespan (since the age of 18 years). Expert health professionals conducted semi-structured interviews to collect appropriate information regarding the retrospective MRIq data, specifically from the age of 18 years to the patients’ age at the time of the cognitive and MRI assessment. The questionnaire contains 17 items that evaluate the frequency of activities that deal with motor range calculated in terms of years of practice from the age of 18 years to the respondent’s current age. The MRIq is grouped into six section subscores: domestic activities (MRIq_DA), distinguished based on the degree of physical effort required (i.e., low, moderate, high); walking activities (MRIq_Walk), comprising short and long distance and the use of stairs; leisure activities (MRIq_LA), comprising leisure time occupations (i.e., outside of working time or school schedule) executed while standing or sitting; physical exercise (MRIq_PE), distinguished based on the degree of physical effort required (i.e., low/moderate, high/agonistic); caring activities (MRIq_CA), comprising caring for children or elderly people; and working activities (MRIq_WA), distinguished based on the degree of physical effort required for executing them (i.e., low, moderate, high), and on working activities executed while standing or requiring walking. An Excel file for automatic calculation of subscores was made available by the authors who conceptualised and developed the MRIq. The calculation allowed us to obtain the percentage of corrected MRIq scores in each subscale and the total percentage index of MR (MRIq_Tot). To determine the percentage, the frequency expressed in terms of years spent to execute the activity was divided by the total age (from 18 years to the age at the time of assessment), and the result was multiplied by 100. Higher MRIq scores were considered to be representative of higher estimated MR.

### 2.3. Motor and Cognitive Measures

The characteristics of cerebellar motor signs in each patient were quantified by an expert neurologist by using the ICARS [26], the global score of which ranges from 0 (absence of any motor deficit) to 100 (presence of motor deficits at the highest degree). The total score of the scale is determined by the sum of the subscores obtained in each section, which evaluate specific ataxic symptoms and comprise the scale as follows: postural and gait disturbances (maximum score: 34), kinetic functions (maximum score: 52), speech disorders (maximum score: 8), and oculomotor disorders (maximum score: 6).

A detailed battery of neuropsychological tests was administered to each subject to assess the following cognitive domains: 

Current intellectual functioning: Wechsler Adult Intelligence Scale—Revised [27,28];

Executive functions: Phonological fluency [29]; Wisconsin Card Sorting Test—perseverative errors [30]; Stroop Test—accuracy score [31,32];

Short-term memory: Immediate recall of Rey’s 15 words [33]; forward and backwards digit span [34,35]; Corsi Test [36]; immediate recall of Short-Story Recall Task [37];

Long-term memory: Delayed recall of Rey’s 15 Words [33]; Delayed recall of Short-Story Recall task [37];

Attention: Multiple Features Target Cancellation task (MFTC)—accuracy scores [38]; Line Cancellation Task—accuracy scores [39];

Processing speed: Stroop Test—execution time [31,32]; Multiple features target cancellation task (MFTC)—execution time [38]; Line Cancellation Task—execution time [39].

### 2.4. MRI Data Protocol

An MRI inspection at 3 T (Magnetom Allegra, Siemens, Erlangen, Germany) was requested for all participants, and included the following acquisitions: (1) dual-echo turbo spin-echo (TSE) (TR = 6190 ms, TE = 12/109 ms); (2) fast-FLAIR (TR = 8170 ms, 204TE = 96 ms, TI = 2100 ms); (3) 3D modified driven equilibrium Fourier transform (MDEFT) scan (TR = 1338 ms, TE = 2.4 ms, matrix = 256 × 224 × 176, in-plane FOV = 250 × 250 mm^2^, slice thickness = 1 mm); (4) T2*-weighted echo-planar imaging (EPI) sensitised to blood-oxygenation-level-dependent imaging (BOLD) contrast (TR: 2080 ms, TE: 30 ms, 32 axial slices parallel to AC-PC line, matrix: 64 × 64, pixel size: 3 × 3 mm^2^, slice thickness: 2.5 mm, flip angle: 70°) for resting state fMRI. During rest, BOLD echo-planar images were acquired for 7 min and 20 s periods, resulting in a total of 220 volumes. For the duration of this acquisition, participants were required not to reflect about anything in particular, to have their eyes closed, and not to fall asleep. Being included in this research, the TSE scans of all patients were acquired and revised by a practiced neuroradiologist to describe the anatomy of the brain and define the occurrence of macroscopic structural anomalies of extracerebellar areas. According to the inclusion criteria, conventional MRI scans were examined for the control subjects as well, in order to rule out any pathological conditions.

#### 2.4.1. Resting-State fMRI Data Pre-Processing

Statistical parametric mapping (Wellcome Department of Imaging Neuroscience; SPM8 (http://www.fil.ion.ucl.ac.uk/spm/)) and in-house software implemented in MATLAB (MathWorks Inc., Natick, MA, USA) were used to pre-process the data. To permit T1 equilibration effects, we rejected the first four volumes of the fMRI series for each subject. The data pre-processing included the following steps: correction for head motion, compensation for slice-dependent time shifts, normalisation to the EPI template in MNI coordinates supplied with SPM8, and smoothing with a 3D Gaussian kernel with 8 mm^3^ full width at half-maximum. The parameters of motion assessed during correction were inspected for every dataset to guarantee that the maximum absolute shift did not exceed 2 mm and the maximum absolute rotation did not exceed 1.5°. The global temporal drift was taken off using a 3rd-order polynomial fit, the signal was regressed against the realignment parameters, and the signal was balanced over whole-brain voxels to eliminate further possible causes of bias. Next, to lessen the consequences of low-frequency drift and high-frequency physiological noise, all images were filtered by a phase-insensitive bandpass filter (pass band 0.01–0.08 Hz).

#### 2.4.2. Network-Based Statistics

A body of 116 nodes determined by the Automated Anatomical Labelling (AAL) Atlas was first defined in order to obtain a connectivity matrix for every subject. The mean time-course of every node was estimated as the average of the fMRI time series from all voxels in a certain region. Then, we obtained correlation matrices by estimating the correlation between all mean signals in the pairs of nodes, as detailed by Serra et al. [40]. Thus, changes in FC between definite cerebellar and cerebral “nodes” were detected. The statistical comparison was carried out by using the NBS tool developed by Zalensky et al. [41]. The comparison of FC matrices between patients and controls was conducted by carrying out a two-sample *t*-test, with 5000 permutations and the statistical significance (*p*-value) set at 0.05, adjusted for multiple comparisons by means of NBS correction [41].

### 2.5. Statistical Analyses

To cluster the different cognitive tests according to the equivalent functional domain, each subject’s raw score was converted into a Z-score, according to the following formula: (subject raw score—population mean score)/population standard deviation (SD). Published normative data were used for the following tests: Rey’s 15 Words, Short Story Test, MFTC, and the Line Cancellation Task. For the remaining tests, the mean scores of the population were obtained from raw scores of specific control groups constituted by individuals who had no history of psychiatric or neurological illness. Statistical analysis revealed no significant differences between SCA2 patients and the healthy subjects of the control groups in terms of mean age and education (*t*-test considered significant at *p* < 0.05). For a detailed report of the demographic and cognitive data of healthy controls, see Table S1 in the Supplementary Materials. Subsequently, composite Z-scores were computed for each cognitive domain by calculating the mean Z-scores of the tests grouped on the basis of the corresponding functional domain.

A correlational analysis was performed by carrying out Spearman’s rank-order correlation test to determine the relationships between individual MRIq scores and (i) age and education, (ii) total score on the ICARS and the individual scores on the ICARS subscales, (iii) performance in each cognitive domain, and (iv) the patterns of increased internodal FC. To confirm the use of Spearman’s test, each scatterplot derived from the association between each pair of variables was inspected to verify whether the relationship appeared monotonic. Statistical analyses were performed using the Statistical Package for the Social Sciences (SPSS) version 25.

## 3. Results

The individual scores of SCA2 patients on the Motor Reserve Index Questionnaire (MRIq) are reported in Table 2. For a detailed report of the ICARS scores of each SCA2 patient, see Table S2 in the Supplementary Materials.

### 3.1. Correlation between MRIq Scores and Motor/Cognitive Measures

**Figure 1:**
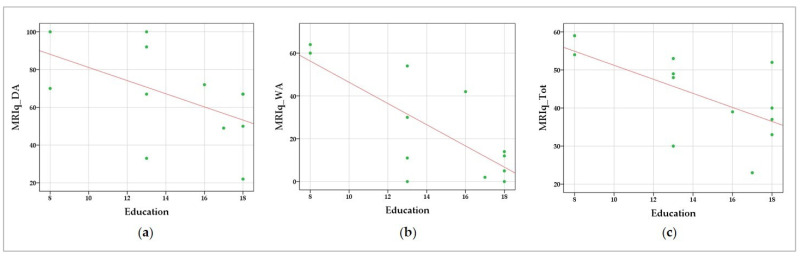
Data scatterplots of significant correlations between education and (**a**) MRIq_DA—domestic activities, (**b**) MRIq_WA—working activities, and (**c**) MRIq_Tot—total score. The green dots indicate the values for an individual data point; the fit lines representing the trend of the data are reported in red.

The correlational analysis performed using Spearman’s test revealed substantial correlations between MRIq variables and many of the measures taken into account. Negative correlations were found between educational levels and MRIq_DA (r = −0.583; *p* = 0.023), MRIq_WA (r = −0.589; *p* = 0.022), and MRIq_Tot (r = −0.559; *p* = 0.029), indicating a relationship between educational level and MR resulting from domestic and working activities requiring physical effort. The results of the correlation analyses are detailed in Table 3. The data scatterplots of significant correlations are shown in Figure 1.

**Figure 2:**
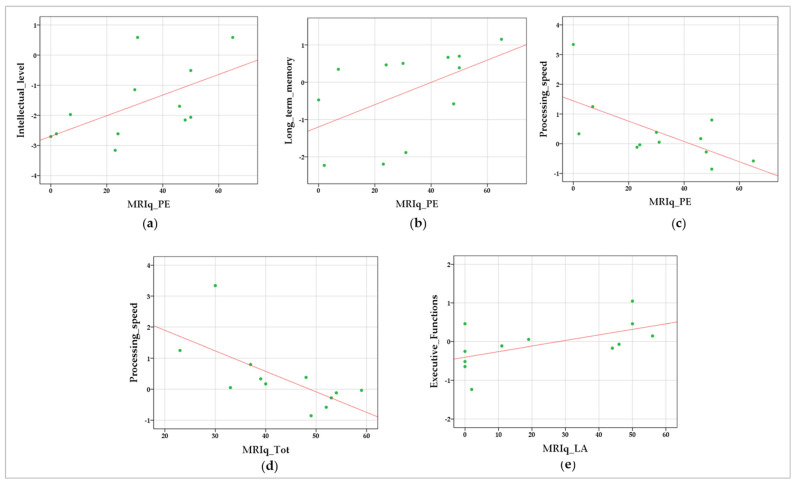
Data scatterplots of significant correlations between MRIq and cognitive domains: Correlations between MRIq_PE—physical exercise and (**a**) intellectual level, (**b**) long-term memory, and (**c**) processing speed; between MRIq_Tot—total score and (**d**) processing time; and between MRIq_LA—leisure activities and (**e**) executive function. The green dots indicate the values for an individual data point; the fit lines representing the trend of the data are reported in red.

Positive correlations were found between MRIq_PE and intellectual level (r = 0.661; *p* = 0.010) and measures of long-term memory (r = 0.630; *p* = 0.028), representative of an association between motor reserve driven by physical exercise and better general cognitive functioning and long-term memory abilities. In addition, a negative correlation was detected between processing speed and both MRIq_PE (r = −0.606; *p* = 0.018) and MRIq_Tot (r = −0.762; *p* = 0.002), revealing a relationship between motor reserve and the ability to process information quickly. Finally, a positive correlation was revealed between MRIq_LA and executive function (r = 0.585; *p* = 0.023), revealing a relation between executive function and leisure activities demanding motor load. The results of the correlation analyses are detailed in Table 3. The data scatterplots of significant correlations are shown in Figure 2.

**Figure 3:**
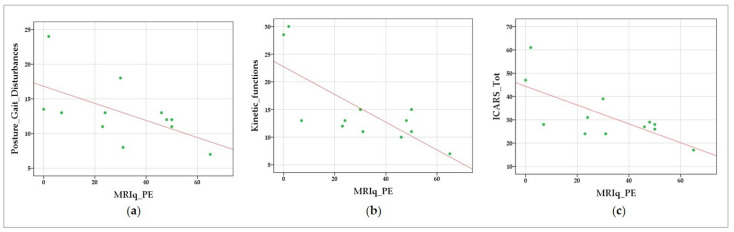
Data scatterplots of significant correlations between MRIq and clinical motor symptoms: Correlations between MRIq_PE—physical exercise and (**a**) posture and gait disturbances, (**b**) kinetic functions, and (**c**) total ICARS scores. The green dots indicate the values for an individual data point; the fit lines representing the trend of the data are reported in red.

Negative correlations were found between MRIq_PE and measures of motor functioning, namely, with posture and gait disturbances (r = −0.673; *p* = 0.008), kinetic functions (r = −0.616; *p* = 0.016), and the total ICARS score (r = −0.603; *p* = 0.019), likely indicating that the longer the time spent on physical exercise during an individual’s lifetime, the lower the severity of symptoms related to standing and walking capacities and to limb and body motion. The results of the correlation analyses are detailed in Table 4. The data scatterplots of significant correlations are shown in Figure 3.

### 3.2. Functional Connectivity Results and Correlation with MRIq

NBS analysis showed increased internodal connectivity between and within cerebellar and cerebral regions throughout the whole brain in SCA2 patients compared to controls. Overall, 16 nodes and 12 edges showed differences in SCA2 brains. In particular, increased FC was found within motor regions of the cerebral cortex—i.e., the right medial frontal cortex (MFC) and bilateral supplementary motor area (SMA)—and motor regions of the cerebellum, i.e., the vermis X and the left anterior lobules IV and V. Detailed results of NBS analysis are reported in Table 5, which shows the edges of significant FC increases involving both the cerebellum and the cerebral cortex in SCA2 patients.

**Figure 4:**
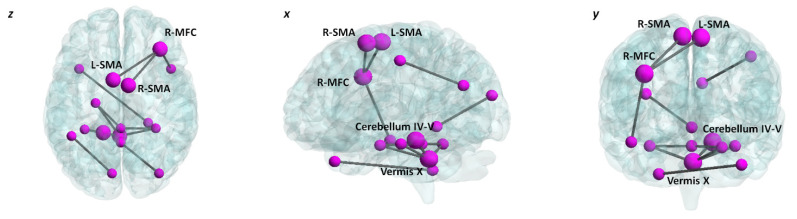
Internodal increased FC patterns: Significantly increased internodal connectivity in SCA2 patients as assessed by NBS analysis (FWE = 0.05) (bigger and smaller nodes). Labels represent bigger nodes that correspond to cerebellar and cortical regions showing significant correlations with MRIq (see Table 6). The brain network is visualised using the BrainNet Viewer (https://www.nitrc.org/projects/bnv/) [42] in axial (z), sagittal (x), and coronal (y) sections. MFC = medial frontal cortex; SMA = supplementary motor area; R = right; L = left.

**Figure 5:**
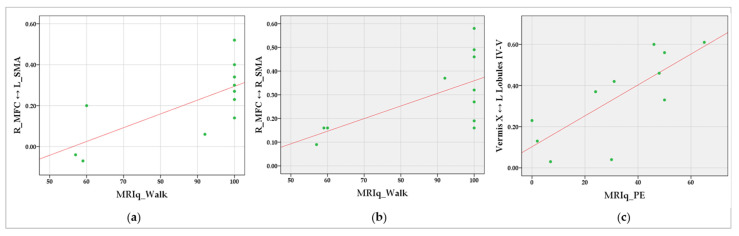
Data scatterplots of significant correlations between MRIq and increased internodal FC between (**a**) the right MFC and the left SMA, (**b**) the right MFC and the right SMA, and (**c**) the vermis X and the left cerebellar anterior lobules (IV–V). The green dots indicate the values for an individual data point; the fit lines representing the trend of the data are reported in red.

Interestingly, significant correlations were found between specific MRIq measures and patterns of increased internodal FC involving motor cerebral and cerebellar regions in SCA2 patients. Specifically, positive correlations were detected between MRIq_Walk and increased internodal FC between the right MFC and the left SMA (r = 0.790; *p* = 0.004), and between the right MFC and the right SMA (r = 0.638; *p* = 0.035). Positive correlations were also reported between MRIq_PE and increased internodal FC within the cerebellum, specifically between cerebellar vermis X and the left cerebellar anterior lobules (IV–V) (r = 0.738; *p* = 0.010). The FC results that significantly correlated with the MRIq scores are detailed in Table 6. Pairwise brain regions showing increased functional connectivity in SCA2 are shown in Figure 4. The data scatterplots of significant correlations are shown in Figure 5.

## 4. Discussion

The notion that a person is wholly healthy when they are both intellectually and physically active has been held since ancient times, and it is archetypally exemplified in the motto “*mens sana in corpore sano*”. A more novel issue is the extent to which pre-existing and enriched cognitive and motor abilities (i.e., cognitive and motor reserve) contribute to coping with neurodegeneration and a decline in these capabilities. Despite the increasing interest in this wide-ranging topic, most research conducted thus far has emphasised cognitive reserve, while very few studies have focused on motor reserve [2,6].

Therefore, this is the first study aimed at estimating MR in a homogenous sample of SCA2 patients based on an ad hoc MR questionnaire, and at exploring the associations between the resulting MR scores, motor and cognitive performances, and patterns of increased FC in cerebral and cerebellar networks. Several major findings were obtained, suggesting a relationship between SCA2 patients’ MR and their ability to cope with ataxia-related symptoms and specific cognitive functions, as well as between MR and patterns of increased internodal FC likely to indicate the identification of MR neural substrates and, thus, the existence of underlying compensatory mechanisms. 

We observed that a higher MR was associated with a lower severity of motor symptoms as assessed by the ICARS. In detail, higher MR in terms of time spent engaged in sports during an individual’s lifetime resulted in a higher chance for patients to preserve their ability to stand and walk, and to move their limbs and body, enhancing their ability to handle ataxic symptoms. Our results are consistent with those obtained in a study conducted on patients with PD, demonstrating that patients who engaged in more physical exercise during the premorbid period showed fewer motor deficits later in life than those who exercised less, despite similar degrees of dopamine reduction [4]. A similar mechanism is stated for cognitive reserve; indeed, AD patients who reported lifelong experiences such as leisure activities and occupational attainment showed increased cognitive reserve and lower susceptibility to brain and cognitive changes associated with the disease [43]. Despite the paucity of studies on MR in general, taken together, our results confirm a protective effect of reserve in terms of physical exercise engagement with respect to disease symptom severity in cerebellar neurodegenerative diseases.

What we refer to as cognitive and motor reserve in human studies partially mirrors the concept of environmental enrichment as accounted for in animal studies, revealing a great effect of housing in new and composite surroundings on improved levels of sensory, motor, and cognitive stimulation in mice and rats [44,45,46,47]. Consistently, we found that higher MR determined by physical exercise was correlated with many of the cognitive domains we took into account, such as patients’ better general cognitive functioning, long-term memory abilities, and processing speed, the latter being also associated with total MR estimates, further supporting the existence of an interaction between motor exercise and cognitive functioning. In line with this, we also found that higher MR driven by the time spent on leisure activities executed while standing or sitting was associated with better performance of SCA2 patients in executive function. Both fine and gross motor skills are required for the implementation of the leisure activities as defined by the MRIq (e.g., knitting, gardening, hunting). Indeed, the physical movements involved in these activities require the implementation of coordinated movements involving the whole body and its individual parts for the execution of the required motor actions that, in turn, need executive functions to be properly performed, such as planning, working memory, and goal-directed actions. Our results are consistent with the well-established intertwined relationship between executive and motor functions, mutually engaged for the early development and management of both functions in ageing, and affecting one another in pathological conditions [48,49,50].

Remarkably, we observed that greater MR driven by domestic and working activities requiring motor effort was associated with a lower educational level. Although this result contrasts with other findings reporting the role of educational attainment in enhancing motor reserve in PD [6], it might indicate a trend of people with a low level of education to engage in working employment that entails activities requiring more physical—rather than intellectual—effort, thus increasing MR.

Interestingly, additional evidence to support the role of MR in SCA2 patients comes from the MRI data. Indeed, the NBS analysis revealed patterns of increased internodal connectivity between specific cerebral and cerebellar areas. Abnormal connectivity within cerebello–cortical regions has been consistently described in SCA2 patients, suggesting that the impaired cerebello–cerebral interaction may explain the widespread deficits typically observed in such patients [15,16,23]. However, while patterns of decreased FC are typically explored and described as representative of deteriorated pathways associated with affected functions, increased FC is described in the literature in terms of both degenerative and compensatory modifications [9,51].

The present study revealed SCA2 patients’ patterns of increased internodal cerebral–cerebellar FC that do not correlate with MRIq measures (see Table 5). Interestingly, alterations in these areas are associated with patients’ deficits in motor, sensorimotor, and cognitive functions [15,23], and have been explained in terms of pathological functional mechanisms related to cerebellar alterations [52]. 

On the other hand, the patterns of increased FC that correlated with MRIq indices (see Table 6) occurred in cerebral and cerebellar nodes specifically involved in motor functions. Thus, we advance the hypothesis that these patterns of increased FC represent MR networks supporting compensatory mechanisms to cope with the disease, and can be described as MR biomarkers. Overall, specific patterns of increased FC might indicate that functional networks reorganise, insofar as they are supported by patients’ behavioural engagement in cognitive and motor activities throughout their lifespan [7].

In detail, the correlations between MR measures defined in terms of walking activities employed throughout a lifetime and increased internodal FC between the right MFC and the left SMA, and between the right MFC and the right SMA, are consistent with the literature on the functions of these areas. The SMA is generally required for the control of movement and, more specifically, the control of postural stability during stance or walking [53], planning and coordination of temporal sequences of movements [54], and self-initiated actions (e.g., rising from a chair, walking), rather than movements that occur in response to an external trigger or stimulus (e.g., catching a ball) [55]. In addition, the interaction between the SMA and frontal regions, such as the MFC, is crucial for motor sequences and action planning [56].

Additionally, the correlation between MR estimates in terms of physical exercise and the increased internodal FC between the cerebellar vermis X and left cerebellar lobules IV–V is consistent with the functional topography of the cerebellum [57]. The vermis X is part of the flocculonodular lobe of the cerebellum—also known as the vestibulocerebellum—and is involved in equilibrium control and postural movements [58]. This lobe plays a key role in controlling the balance between agonist and antagonist muscle contractions of the spine, hips, and shoulders during rapid changes in body position, and during rapid changes in the directions of movements [58]. Lobules IV–V of the anterior cerebellum are known to be coupled with sensorimotor circuits in support of motor execution [57]. These cerebellar regions are mostly active during hand movements, stimulation of the hands and feet, and while performing tasks with motor planning demands, as well as when learning and planning stimulus-driven movements and actions [59,60,61]. Considering all of these features, it is not surprising that a higher internodal connectivity within these areas is associated with higher MR in terms of physical exercises required for sporting implementations. Our results are consistent with evidence on the role of animal environmental enrichment in increasing cerebellar compensation and promoting cerebellar reserve [62].

Overall, the identified networks of increased internodal FC that were correlated with the MRIq indices might reflect a biomarker likely to underlie lifetime MR in SCA2 patients, thus impacting the severity of motor symptoms. It needs to be underlined that, although intriguing, our assumptions related to network reorganisation should be verified by detecting FC patterns in premorbid states of the disease to verify whether they represent networks that compensate for the pathology associated with enhanced MR. Additionally, MR is a relatively novel matter of interest; as such, the conclusions that we have drawn from our results are speculative in nature, and need to be interpreted with caution and replicated in the future. Nevertheless, the uniformity of our sample of SCA2 patients—a rare disease with specific genetic roots—allowed us to obtain definite and consistent correlational results despite the small sample size. Importantly, given the genetic nature of SCA2, the early assessment of the risk of developing the disease, together with early measurement of MR, could have a factual impact and utility in the clinical setting, eliciting clinical treatments for both morbid and premorbid status, and likely having an effect on disease progression. Hence, a quantification of MR in early stages and at different time points, as assessed by specific tools such as the MRIq, might help to implement precocious and individualised interventions that boost the benefits and improve patients’ quality of life.

## Figures and Tables

**Table 1:** Demographic characteristics of SCA2 patients.

ID	Age	Education (Years)	M/F	CAG	ICARS	Disease Duration (Months)
CB1	42	13	F	35 ± 1	47	7
CB2	40	18	F	47 ± 1	26	147
CB3	64	17	M	35 ± 1	28	42
CB4	54	18	F	37 ± 1	27	45
CB5	60	8	F	37	31	42
CB6	43	13	F	n.a.	28	154
CB7	38	13	F	42	39	114
CB8	42	18	M	39	17	64
CB9	54	18	M	n.a.	24	n.a.
CB10	48	13	M	38 ± 1	29	47
CB11	51	8	M	37	24	156
CB12	44	16	F	n.a.	61	118
Means (SD)	48.33 (8.29)	14.42 (3.70)	5/7	-	31.75 (11.91)	85.09 (53.71)

CAG = Number of expanded triplets. n.a.: CAG size data are not available for patients CB29, CB40, CB49 because at the time of the diagnosis, genetic testing did not include determination of the number of triplet repeats. F = female; M = male; ICARS = International Cooperative Ataxia Rating Scale. ICARS range: minimum score 0 (absence of motor deficits), maximum score 100 (maximum presence of motor deficits). Disease duration represents the time period from the genetic testing.

**Table 2:** Individual results of the Motor Reserve Index Questionnaire (MRIq) in SCA2 patients.

ID	MRIq—DA	MRIq—Walk	MRIq—LA	MRIq—PE	MRIq—CA	MRIq—WA	MRIq—TOT (%)
CB1	33	92	46	0	0	11	30
CB2	50	100	0	50	16	5	37
CB3	49	59	0	7	21	2	23
CB4	67	100	11	46	15	0	40
CB5	100	100	19	24	45	64	59
CB6	92	60	44	50	46	0	49
CB7	100	100	0	30	28	30	48
CB8	67	100	56	65	13	12	52
CB9	22	57	0	31	72	14	33
CB10	67	100	50	48	0	54	53
CB11	70	100	50	23	23	60	54
CB12	72	100	2	2	15	42	39
Means	65.75	89	23.17	31.33	24.5	24.5	43.08
(SD)	(24.52)	(18.44)	(23.81)	(21.07)	(20.78)	(24.43)	(11.07)

Corrected MRIq scores in each subscale and the total percentage index of motor reserve are reported. MRIq—DA = MRIq—domestic activities; MRIq—Walk = MRIq—walking activities; MRIq—LA = MRIq—leisure activities; MRIq—PE = MRIq—physical exercise; MRIq—CA = MRIq—care activities; MRIq—WA = MRIq—work activities; MRIq—TOT = MRIq—total percentage score.

**Table 3:** Correlations between Motor Reserve Index Questionnaire (MRIq) scores and cognitive variables.

	MRIq—DA	MRIq—Walk	MRIq—LA	MRIq—PE	MRIq—CA	MRIq—WA	MRIq—TOT (%)
Education	***R*** **= *−0.58;*** ***p* = *0.02***	R = −0.17; *p* = 0.30	R = −0.37; *p* = 0.12	R = 0.42; *p* = 0.08	R = −0.16; *p* = 0.32	***R*** **= *−0.59;*** ***p* = *0.02***	***R*** **= *−0.56;*** ***p* = *0.03***
Intellectual level	R = −0.12; *p* = 0.36	R = −0.33; *p* = 0.15	R = −0.25; *p* = 0.22	***R*** **= *0.66;*** ***p* = *0.01***	R = 0.33; *p* = 0.15	R = −0.49; *p* = 0.06	R = −0.16; *p* = 0.31
Executive functions	R = −0.14; *p* = 0.33	R = 0.40; *p* = 0.10	R = 0.59; *p* = 0.02	R = 0.44; *p* = 0.08	R = −0.37; *p* = 0.12	R = 0.21; *p* = 0.25	R = 0.48; *p* = 0.06
Short-term memory	R = −0.11; *p* = −74	R = −0.38; *p* = 0.22	R = 0.12; *p* = 0.72	R = 0.56; *p* = 0.06	R = 0.34; *p* = 0.28	R = −0.22; *p* = 0.50	R = 0.15; *p* = 0.63
Long-term memory	R = −0.27; *p* = −39	R = 0.10; *p* = 0.77	R = 0.12; *p* = 0.72	***R*** **= *0.63;*** ** = *0.03***	R = 0.07; *p* = 0.83	R = −0.51; *p* = 0.09	R = 0.20; *p* = 0.54
Attention	R = −0.33; *p* = 0.19	R = −0.29; *p* = 0.23	R = −0.29; *p* = 0.23	R = 0.30; *p* = 0.22	R = 0.09; *p* = 0.41	R = −0.29; *p* = 0.22	R = −0.26; *p* = 0.25
Processing speed	R = −0.40; *p* = 0.10	R = −0.17; *p* = 0.30	R = −0.62; *p* = 0.02	***R*** **= *−0.61;*** ***p* = *0.02***	R = −0.20; *p* = 0.27	R = −0.21; *p* = 0.26	***R*** **= *−0.76;*** ***p* = *0.00***

Correlations significant at *p* < 0.05 are presented in bold and italic type. MRIq—DA = MRIq—domestic activities; MRIq—Walk = MRIq—walking activities; MRIq—LA = MRIq—leisure activities; MRIq—PE = MRIq—physical exercise; MRIq—CA = MRIq—care activities; MRIq—WA = MRIq—work activities; MRIq—TOT = MRIq—total percentage score.

**Table 4:** Correlations between Motor Reserve Index Questionnaire (MRIq) scores and International Cooperative Ataxia Rating Scale (ICARS) scores.

	MRIq—DA	MRIq—Walk	MRIq—LA	MRIq—PE	MRIq—CA	MRIq—WA	MRIq—TOT (%)
Posture and gait disturbances	R = 0.27; *p* = 0.20	R = 0.21; *p* = 0.26	R = −0.38; *p* = 0.11	***R*** **= *−0.67;*** ***p* = *0.01***	R = −0.23; *p* = 0.24	R = 0.08; *p* = 0.40	R = −0.28; *p* = 0.19
Kinetic functions	R = 0.33; *p* = 0.15	R = −0.15; *p* = 0.32	R = −0.10; *p* = 0.38	***R*** **= *−0.62;*** ***p* = *0.02***	R = −0.01; *p* = 0.48	R = 0.17; *p* = 0.30	R = −0.15; *p* = 0.32
Speech disorders	R = 0.10; *p* = 0.38	R = 0.11; *p* = 0.37	R = −0.24; *p* = 0.23	R = −0.36; *p* = 0.13	R = −0.18; *p* = 0.29	R = 0.25; *p* = 0.22	R = −0.14; *p* = 0.33
Oculomotor disorders	R = 0.18; *p* = 0.29	R = 0.21; *p* = 0.25	R = −0.23; *p* = 0.24	R = −0.41; *p* = 0.10	R = 0.07; *p* = 0.42	R = 0.53; *p* = 0.04	R = 0.00; *p* = 0.50
ICARS TOT	R = 0.34; *p* = 0.14	R = 0.08; *p* = 0.40	R = −0.17; *p* = 0.30	***R*** **= *−0.60;*** ***p* = *0.02***	R = −0.20; *p* = 0.27	R = 0.20; *p* = 0.27	R = −0.13; *p* = 0.34

Correlations significant at *p* < 0.05 are presented in bold and italic type. MRIq—DA = MRIq—domestic activities; MRIq—Walk = MRIq—walking activities; MRIq—LA = MRIq—leisure activities; MRIq—PE = MRIq—physical exercise; MRIq—CA = MRIq—care activities; MRIq—WA = MRIq—work activities; MRIq—TOT = MRIq—total percentage score.

**Table 5:** Pairwise nodes of increased FC in SCA2 patients.

Pairwise Brain Regions		*t*-Values
Nodes within the cerebral cortex		
R MFC	L SMA	4.50
	R SMA	4.28
	R superior temporal pole	4.26
L Cuneus	L inferior parietal cortex	4.53
Cerebellar-cerebral nodes		
Vermis I–II	L parahippocampal cortex	4.04
	R fusiform cortex	4.09
Vermis IV–V	R occipital medial cortex	4.00
Vermis X	L parahippocampal cortex	3.95
	R fusiform cortex	4.39
	L fusiform cortex	4.44
R lobule X	L medial temporal pole	3.82
Nodes within the cerebellum		
Vermis X	L lobules IV–V	3.64

Functional connectivity between pairwise cerebellar and cerebral regions in patients with SCA2 compared with controls (*p*-value < 0.05 after FDR correction using network-based statistics); R = right; L = left; MFC = medial frontal cortex; SMA = supplementary motor area.

**Table 6:** Increased internodal FC patterns that significantly correlated with MRIq scores.

Increased Internodal FC	MRIq—DA	MRIq—Walk	MRIq—LA	MRIq—PE	MRIq—CA	MRIq—WA	MRIq—TOT (%)
R MFC ↔ L SMA	R = 0.33; *p* = 0.33	***R *** **= * 0.79;*** ***p* = *0.00***	R = 0.14; *p* = 0.69	R = 0.52; *p* = 0.10	R = −0.36; *p* = 0.28	R = 0.01; *p* = 0.99	R = 0.34; *p* = 0.31
R MFC ↔ R SMA	R = −0.10; *p* = 0.77	***R *** **= * 0.64;*** ***p* = *0.04***	R = 0.39; *p* = 0.23	R = 0.34; *p* = 0.31	R = −0.82; *p* = 0.00	R = 0.00; *p* = 1	R = 0.14; *p* = 0.69
Vermis X ↔ L lobules IV–V	R = 0.14; *p* = 0.69	R = 0.38; *p* = 0.25	R = 0.42; *p* = 0.19	***R*** **= *0.74;*** ***p* = *0.01***	R = −0.26; *p* = 0.44	R = −0.10; *p* = 0.78	R = 0.40; *p* = 0.22

Correlations significant at *p* < 0.05 are presented in bold and italic type. MRIq—DA = MRIq—domestic activities; MRIq—Walk = MRIq—walking activities; MRIq—PE = MRIq—physical exercise; MRIq—CA = MRIq—care activities; MRIq—WA = MRIq—work activities; MRIq—TOT = MRIq—total percentage score; R = right; L = left; MFC = medial frontal cortex; SMA = supplementary motor area.

## Data Availability

The data presented in this study are available on request from the corresponding author on reasonable request.

## Supplementary Materials

The following are available online at https://www.mdpi.com/article/10.3390/biomedicines10092166/s1, Table S1: Age, education, and performances of the different control groups employed; Table S2: Individual scores on the International Cooperative Ataxia Rating Scale (ICARS) for SCA2 patients.
