# Supplementary material for: “Mens Sana in Corpore Sano”: The Emerging Link of Motor Reserve with Motor and Cognitive Abilities and Compensatory Brain Networks in SCA2 Patients
Source: Biomedicines. 2022 Sep 2;10(9):2166. doi: 10.3390/biomedicines10092166 (PMC9496032; doi:10.3390/biomedicines10092166)
Supplement: Supplementary file 1 [file biomedicines-10-02166-s001.zip › Supplementary materials.pdf]

Table S1. Age, education and performances of the different control groups employed.

	Test	n°	Age (years)	Education	Raw score
Intellectual level	QI WAIS-r	32	42,42 (9,51)	12,03 (3,8)	103,57 (10,76)
Executive functions	Phonological fluency	72	48,14 (12,70)	13,42 (3,66)	40,77 (10,18)
	WCST n° perseverative errors	43	47,44 (12,11)	13,91 (3,48)	7,95 (6,85)
	Stroop Test - accuracy	43	47,44 (12,11)	13,91 (3,48)	0,31 (1,57)
Shot-term memory	Rey's 15 words – Immediate recall *	340	53,1 (18,00)	10,2 (4,30)	42,31 (10,16)
	Forward digit span	63	43,56 (14,58)	12,17 (3,19)	6,08 (1,24)
	Backward digit span	63	43,56 (14,58)	12,17 (3,19)	4,45 (0,97)
	Forward Corsi	63	51,13 (15,49)	12,33 (4,12)	5,82 (1,19)
	Short-Story Recall - Immediate recall *	30	40 – 49	13,3 (3,4)	5,95 (1,38)
Long-term memory	Rey's 15 words – Delayed recall *	340	53,1 (18,00)	10,2 (4,30)	8,90 (3,16)
	Short-Story Recall - Delayed recall *	30	40 – 49	13,3 (3,4)	5,9 (1,45)
Attention	MFTC - Accuracy *	465	62,1 (20,96)	8,92 (5,32)	0,94 (0,058)
	Lines cancellation task – Accuracy *	40	69,3 (8,0)	8,4 (4,2)	59,80 (0,40)
Processing speed	Stroop Test – execution time	43	47,44 (12,11)	13,91 (3,48)	19,17 (10,00)
	MFTC - Execution time *	465	62,1 (20,96)	8,92 (5,32)	80,95 (40,07)
	Lines cancellation task – Execution time *	40	69,3 (8,0)	8,4 (4,2)	47,30 (24,50)

* Published normative data

Table S2. Individual scores of International Cooperative Ataxia Rating Scale (ICARS) in SCA2 patients.

ID	Posture and Gait Disturbances	Kinetic Functions	Speech Disorders	Oculomotor Disorders	ICARS TOT
**CB1**	13,5	28,5	3	2	47
**CB2**	12	11	2	1	26
**CB3**	13	13	2	0	28
**CB4**	13	10	3	1	27
**CB5**	13	13	3	2	31
**CB6**	11	15	2	0	28
**CB7**	18	15	3	3	39
**CB8**	7	7	2	1	17
**CB9**	8	11	3	2	24
**CB10**	12	13	3	1	29
**CB11**	11	12	0	1	24
**CB12**	24	30	5	2	61
**Means (SD)**	12,96 (4,44)	14,87 (7,06)	2,58 (1,16)	1,33 (0,89)	31,75 (11,95)

ICARS range: minimum score 0 (absence of motor deficits), maximum score 100 (maximum presence of motor deficits). Postural and gait disturbances: maximum score 34; kinetic functions: maximum score 52; speech disorders: maximum score 8; oculomotor disorders: maximum score 8.
